# A Miniaturized Sample Preparation Method for the Determination of Vitamins A and E in Food Products

**DOI:** 10.3390/molecules28083449

**Published:** 2023-04-13

**Authors:** Magdalena Słowik-Borowiec, Natalia Głąb, Sabina Stach, Ewa Szpyrka

**Affiliations:** Institute of Biotechnology, Collegium of Natural Sciences, University of Rzeszow, 1 Pigoń St., 35-310 Rzeszów, Poland

**Keywords:** vitamin A, vitamin E, food products, validation, liquid chromatography, HPLC-UV-VIS/DAD

## Abstract

A new analytical approach to the simultaneous identification and quantification of vitamins A and E in three representative matrices (Parmesan, spinach, and almonds) was developed. The analyses were based on high-performance liquid chromatography with UV-VIS/DAD detection. The procedure was optimized by a significant reduction in the weight of the tested products and quantities of reagents added during the saponification and extraction stages. A full method validation study was performed for retinol at two concentration levels (LOQ and 200 × LOQ), which showed satisfactory results, with recoveries ranging from 98.8 to 110.1%, and an average CV of 8.9%. Linearity was tested in the range of 1–500 µg/mL and showed the coefficient of determination R^2^ = 0.999. The satisfactory recovery and precision parameters were achieved for α-tocopherol (LOQ and 500 × LOQ) in the range of 70.6–143.2%, with a mean CV equal to 6.5%. The observed linearity for this analyte in the concentration range of 1.06–532.0 µg/mL was R^2^ = 0.999. The average extended uncertainties were estimated, using a top–down approach of 15.9% and 17.6% for vitamin E and A, respectively. Finally, the method was successfully applied to determine vitamins in 15 commercial samples.

## 1. Introduction

Vitamins are the foundation of our life. These are organic compounds with a low molecular weight that belong to xenobiotic compounds for many living beings, both animals and humans [1]. They should be supplied in small amounts with food or as supplements because they are necessary, for example, for the proper course of a number of metabolic processes and for controlling important reactions in the body. Unlike most nutrients, these compounds do not appear as energy substrates but are often used for the synthesis of coenzymes [2,3].

**Figure 1:**
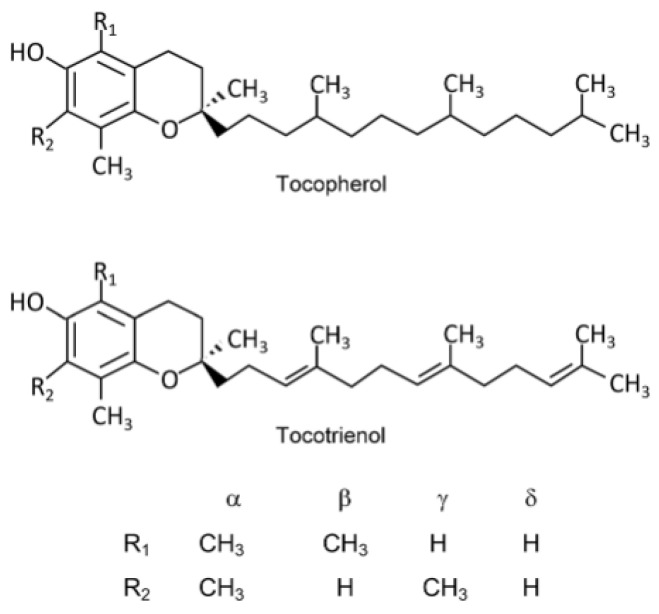
Structures of compounds belonging to the vitamin E group. Reprinted/adapted with permission from Ref. [5]. Copyright 2019, copyright Niki, E.; Abe, K.

The term vitamin E covers a group of eight lipophilic, naturally occurring compounds, including four tocopherols and four tocotrienols. They are designated as α-, β-, γ- and δ- [4]. Tocopherols and tocotrienols share a common core structure. It is composed of a methyl-substituted 6-chromanol benzopyran ring system and an isoprenoid side chain, which in the case of tocopherols is a saturated phytyl tail with three stereocenters at C2, C4′, and C8′. Figure 1 shows the structures of compounds belonging to the vitamin E group.

Tocopherol biosynthesis, which occurs only in photosynthetic organisms (where it is present in all green tissues and seeds), is enantiomerically specific, hence the side chain of naturally occurring tocopherols has the RRR configuration. On the other hand, synthetic tocopherols contain a racemic side chain (usually in the form of all-racemic mixtures) [6,7,8]. Vitamin E belongs to the group of fat-soluble vitamins, which allows it to be stored in the fatty tissues of humans and animals [9]. It is very sensitive to temperature, light, and alkaline conditions. Vitamin E is defined as a biological antioxidant. This action consists in breaking chains, scavenging lipid peroxide radicals, donating hydrogen atoms, and reacting with reactive oxygen and nitrogen species, preventing their excessive accumulation and protecting the entire system against numerous diseases and conditions associated with oxidation, such as cancer, aging, or arthritis. Vitamin E, by preventing lipid peroxidation, protects against the effects of polyunsaturated fatty acids (PUFA), prevents platelet hyper-aggregation, strengthens blood vessel walls, protects the body against atherosclerosis, coronary artery disease, and other cardiovascular diseases, and is involved in the synthesis of hormones [5,9]. Vitamin E is found in various food products, with vegetable oils being the richest source of this vitamin, because they contain all its homologs in various proportions [9]. Nuts and seeds are also good sources. Especially almonds and hazelnuts contain large amounts of α-tocopherol and significant amounts are also found in green leafy vegetables. Additionally, vitamin E is also found in margarine, peanut butter, mango, avocado, asparagus, and, in smaller amounts, in fish, animal liver, eggs, and milk [10,11]. The recommended daily requirement for vitamin E has been established at 11–13 mg of α-tocopherol equivalent (11 mg for adult women and 13 mg for men), while in children it ranges from 5 to 9 mg/day regardless of gender according to the literature data, depending on many factors [12].

**Figure 2:**
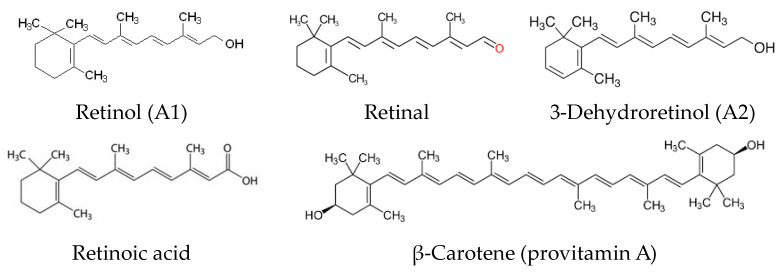
Structures of compounds belonging to the vitamin A group.

Vitamin A is a term referring to several chemical compounds belonging to the group of carotenoids, including retinol and its derivatives and beta-carotene. All forms of vitamin A include substances belonging to polyenes, so they have a similar structure and have a β-ionone ring configuration [13]. There are three main forms of vitamin A, apart from retinol (vitamin A1), derived from animal products, i.e., retinoic acid, retinal, and 3-dehydroretinol (vitamin A2), as well as carotenoids (provitamin A), of plant origin. Formulas of compounds belonging to the vitamin A group are shown in Figure 2 [14,15].

As this vitamin belongs to fat-soluble vitamins, its absorption in the body is facilitated by fats. Compounds containing iron, copper, and oxygen have a destructive effect on it, and it is resistant to high temperatures (up to 130 °C). The oxidation products of vitamin A and carotenes are not biologically active [2,3,16]. There are two main sources of vitamin A. One is foods of animal origin, such as liver, fatty fish, egg yolk, milk, ripened cheeses, and butter. A good source of retinol is meat products (55%), followed by animal fats (about 23%), milk and dairy products (about 11%), and food of plant origin: carrots, pumpkin, peach, apricot, chard, spinach, papaya, mango, parsley leaves, broccoli, kale, and lettuce. 80% of provitamin A is supplied by vegetables and only 5% by fruit [2,16,17,18,19]. Vitamin A has numerous physiological functions, playing important roles in living organisms, such as participating in a process of seeing, e.g., in poor lighting conditions. Rhodopsin, found in the receptors of the retina in the eye, is a light-sensitive protein containing vitamin A [14,20]. Photon-induced isomerization of 11-cis-retinal into all-trans-retinal is an initial stage triggering a series of photochemical events culminating in the optic nerve signaling [21]. Vitamin A and its derivatives have a positive effect on embryogenesis, reproduction, growth and cell differentiation regulation, and the immune system. Retinoids also play an important regulatory role in the skin, by regulating and controlling processes of epithelial keratinization and divisions of skin cells. Thus, they found use in the treatment of skin disorders, such as ichthyosis, psoriasis, or acne [22]. Vitamin A is successfully used for the treatment of cancers (e.g., liver, lungs, breast, and prostate cancers, and melanoma) and measles. Carotenoids, due to their anti-oxidative properties, are also widely used in cosmetics as substances moisturizing the skin and protecting it against UV radiation [15]. The need to balance this vitamin varies, depending on the person’s age, gender, and physical condition. The recommended daily intake (RDA) is set at the level of 900 µg for men and 700 µg for women (adults) and of 300 to 600 µg for younger people, regardless of their gender [23].

The process of determining vitamins A and E in food involves several successive steps, such as saponification, extraction, and identification and quantification of the test substance. When choosing a test technique, important requirements should be taken into account, and, in particular, the type of product tested and the type of vitamin and its concentration in the sample [15,24].

Extraction methods include processes such as esterification, saponification, liquid-liquid extraction, crystallization, and distillation. Combinations of these techniques are usually used to obtain fractions of the highest purity [25,26]. The choice of the method can be determined by the lipid composition of the food. In the case of low lipid concentration, direct extraction is possible, but, when their concentration is high, the saponification process should be performed to destroy structures to which vitamins bind or to eliminate interferences caused by proteins and carbohydrates. The original physicochemical properties of vitamins remain unchanged through the formation of the so-called fraction of unsaponifiable compounds, so their further extraction with organic solvents is possible. The potassium hydroxide (KOH) solution is commonly used for saponification, usually accompanied by an antioxidant, i.e., ascorbic acid, hydroquinone, or pyrogallol and absolute alcohol [27,28]. The saponification process is carried out either at room [29], or more often, at elevated (30–80 °C) temperatures using an ultrasound water bath or a standard water bath, setting the appropriate reaction time as 7 min to even 2 h [30,31,32,33]. Enzymatic hydrolysis represents an alternative to saponification, and it may provide better recoveries [28]. Saponification is usually followed by extraction, which is an important step leading to the separation of the desired compounds from the remaining components of the sample [34]. In the analysis of fat-soluble vitamins, the most frequently used methods of extraction include: liquid–liquid extraction, solid phase extraction, supercritical fluid extraction, pressurized liquid extraction, or microwave-assisted extraction, as techniques alternative to conventional methods [35,36,37]. Among the known instrumental techniques for determining the final concentration of vitamins A and E, gas chromatography or capillary electrochromatography is used, but the most popular method is high-performance liquid chromatography using an ultraviolet-visible (UV-VIS) detector, diode array detection (DAD), fluorescence (FL), or mass spectrometry (MS) [27,34,38].

The methods described in the literature, which are used for the identification and quantification of vitamins A and E, have undeniable advantages, particularly their accuracy, but also have significant disadvantages, especially in terms of time needed and high costs. Therefore, a search continues for new, faster, more efficient, and “green” analytical methods that could be used for routine analyses of these substances [34].

The aim of this study was to modify the method for simultaneous determination of vitamins A and E in food samples using liquid chromatography with a photo-diode array detection (UV-VIS/DAD). The saponification and extraction stages were optimized. A detailed method validation study was conducted to assess its fitness for the specified purpose. Additionally, the optimized method was used to test real food samples for the vitamins A and E content.

## 2. Results

### 2.1. Method Optimization

For the chromatographic separation of vitamins A and E, an ACE 5 RP-C18 column packed with 5 μm (250 mm × 4.6 mm) shell particles was used at a column oven temperature of 30 °C. The mobile phase consisted of methanol (A) and water (B) at a flow rate of 1.0 mL/min, the isocratic elution for 16 min. The injection volume was 20 µL. The first system used A:B solvents (86:14), the second system used a different A:B ratio (95:5), whereas the third isocratic system used 100% solvent A. All elution options were repeated using a 25 °C column oven and a flow rate of 1.2 mL/min. Following the chromatographic analysis, the optimum results with well-separated symmetric peaks of vitamins were obtained for isocratic system of methanol (100%), at a temperature of 30 °C, the flow rate of 1.0 mL/min, and a run time of 16 min. The HPLC-UV-VIS/DAD chromatogram of the stock solution of mixed standards and absorption spectra for retinol and α-tocopherol can be found in the Supplementary Material (Figure S1) attached to this article.

At the saponification stage, 0.5 g of the sample was treated with 50% potassium hydroxide (1.5 mL), methanol (5 mL) with ascorbic acid (0.1 g) added as an antioxidant, and then various approaches to the sample treatment were tested (i–iv) (details are described in Section 4.3. Sample preparation procedure). The saponified sample extracts were transferred to a separatory funnel and extracted three times with hexane (10 mL portions) and washed with water (3 × 20 mL) to remove residual potassium hydroxide. The hexane extracts were dried over anhydrous sodium sulfate. The sample was then concentrated by evaporation to dryness (in N_2_) and the solvent was changed to methanol. The saponification and extraction effectiveness was checked on the basis of the concentration of individual vitamins in the matrices. In the first approach (i), the light-protected samples were left to saponify overnight (18 h) at room temperature (21 °C). The average recovery was 98.8–110.1% for vitamin A and 70.6–143.2% for vitamin E. In the case of (ii), where the samples were placed in the water bath at 80 °C for 30 min, the recovery was 27.8–59.2% and 40.5–689.9% for vitamins A and E, respectively. While in method (iii), with the ultrasound water bath at 60 °C for 25 min, the average recovery was 25.2–77.3% for vitamin A and 61.7–98.0% for vitamin E. In the last case (iv), where the ultrasound water bath at 74 °C for 70 min was used, recoveries were 12.7–76.7% (vitamin A) and 45.0–62.2% (vitamin E). In approach (ii), the recovery rate of 689.9% for Parmesan cheese at the 1 µg/mL fortification level may be associated with chromatographic assay interference; however, the recovery rate at the higher level was also unacceptable at 40.5%. In turn in variant (iii), the parameters for vitamin E were good, while for A, the recovery was significantly lower (from 25%), hence the approach of longer sample preparation but without high temperature was chosen.

### 2.2. Method Validation

The most promising approach to the simultaneous determination of vitamins A and E in food samples turned out to be method (i) the smallest analyte losses were observed during the preparation of the sample extracts. Full method validation was carried out for this process. Validation studies were performed for three representative matrices: Parmesan cheese, spinach, and almonds, at two spiking levels (LOQ and 200 × LOQ (retinol), and LOQ and 500 × LOQ (α-tocopherol). Validation of the analytical method for the vitamin E determination was carried out in accordance with the FDA (U.S. Food and Drug Administration) guidelines for the validation of bioanalytical methods and the ICH technical requirements for the registration of medicinal products for human use [39,40].

Linearity was evaluated by studying six-level calibration curves plotted for retinol and α-tocopherol standards over a concentration range of 1.00–200 μg/mL (vitamin A) and 1.06 to 532 μg/mL (vitamin E), and the following regression equations were obtained: y = 0.5786x + 0.6625 and y = 0.0631x − 0.1524 for vitamins A and E, respectively. The detector response linearity was established using the square determination coefficient (R^2^) determined for the calibration curves, and, at the same time, it was demonstrated that the regression line was linear within the range of tested concentrations (R^2^ = 0.999).

LOD of 0.5 µg/g was obtained for both analytes. LOQs were set at the lowest spiking concentrations, and they were verified by recovery tests (n = 3) and the estimation of the coefficient of variation (CV) for the obtained results in the tested matrices. Satisfactory recovery and precision parameters were obtained for validated vitamins at this lowest level. The LOQ value was 1.00 µg/g for both vitamins.

The working range of the method was established by determining the LOQ and the highest level of spiking, for which acceptance criteria for accuracy, precision, and linearity were met.

**Figure 3:**
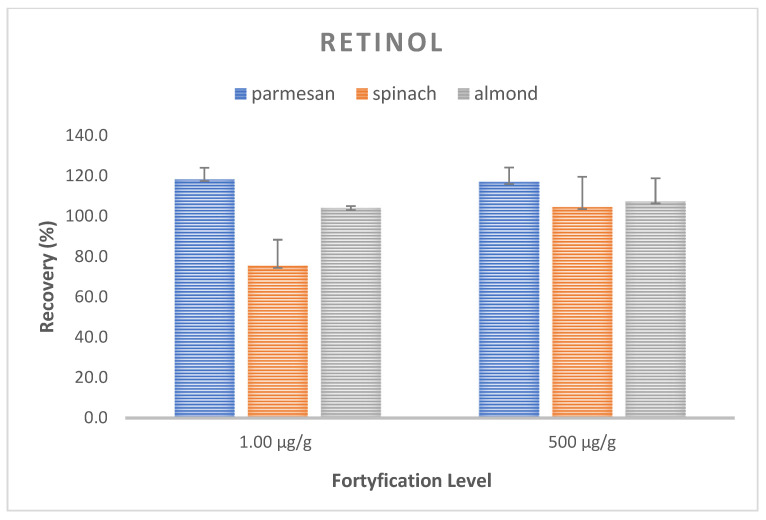
Recovery and CV values for vitamin A at two spiking levels for a matrix of Parmesan, spinach, and almonds.

**Figure 4:**
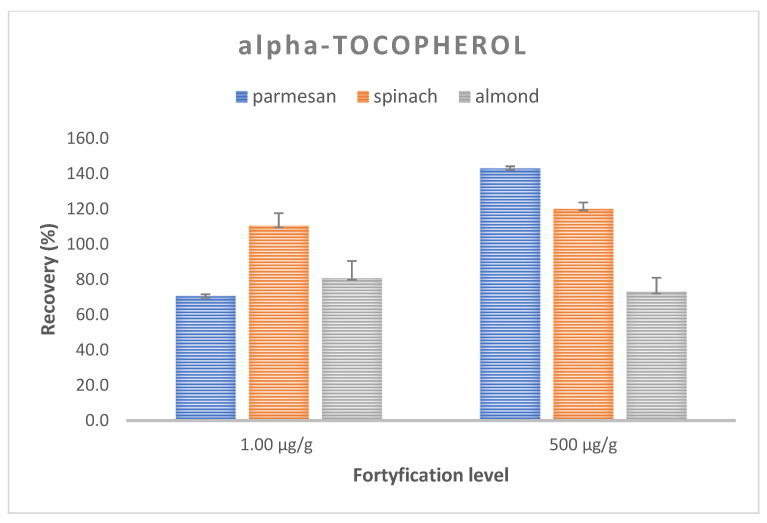
Recovery and CV values for vitamin E at two spiking levels for a matrix of Parmesan, spinach, and almonds.

Trueness and precision expressed as the mean recovery and CV were evaluated for the optimized method by performing recovery experiments at two concentrations. The analysis of the spiked samples was performed in three replications at each level. The mean recovery of retinol in the samples ranged from 98.8–110.1%, with an average CV of 8.9% (117.0–118.4%, CV 5.7–7.1% for the Parmesan matrix; 75.4–104.6%; CV 13.0–15.0% for spinach; and 104.1–107,4%, CV 1.0–11.5% for almonds) (Figure 3). On the other hand, for alfa-tocopherol, the average recovery ranged from 70.6 to 143.2% with the mean CV equal to 6.5%, with 70.6–143.2.7%, CV 5.2–5.7% for the Parmesan matrix; 110.5–120.2%, CV 3.5–7.0% for spinach; and 73.0–80.9%, CV 7.9–9.6% for almonds. These values met the requirements of the FDA and ICH recommendations [39,40], with the recovery ranging from 70 to 120%, with a coefficient of variation (CV) of ≤15% (Figure 4).

Repeatability and recovery proved to be the most important sources of measurement uncertainty, according to the “top–down” experimental approach [41,42,43] in the conducted study. The remaining components of the uncertainty budget, such as uncertainty related to weighing, dilution of standards, and purity of standards, were <5% in the optimized method. The expanded uncertainty in the tested method ranges from 2.6 to 30.5%, with an average of 17.6%, and from 7.2 to 26.4% with an average uncertainty of 15.9%, for vitamins A and E, respectively.

### 2.3. Real Samples

In order to demonstrate the suitability of the optimized method for routine quantifications, real samples were screened for vitamins A and E. The applied methodology is presented in points 4.2–4.4. The UV-VIS/DAD detection was monitored at 325 nm for retinol, and 290 nm for α-tocopherol. Identification of resolved peaks in a real sample was made by comparing their spectra with those derived from the standard solution. The study included 15 samples of food products belonging to similar groups as the tested matrices, i.e., spinach (n = 2), arugula (n = 1), dill (n = 1), parsley (n = 1), cheese (n = 3), mozzarella (n = 1), Parmesan (n = 1), walnuts (n = 2), almonds (n = 1), cashew (n = 1), and pumpkin seeds (n = 1) (Table 1), which were purchased at local commercial shops. The obtained results were compared to literature data (data on the vitamins A and E content in individual products are presented in Table 1). Examples of chromatograms of real samples are presented in the supplementary material (Figure S2).

Retinol was detected in almost all tested samples, with the exception of arugula, fresh spinach, dill, and pumpkin seeds, while α-tocopherol was found in all tested products. The results of vitamins A and E analyses were interpreted by comparing the obtained values to the concentration values for both vitamins provided in the available literature [19,44].

## 3. Discussion

The aim of the work was to demonstrate the usefulness of the developed alternative method for the quantitative determination of retinol and α-tocopherol in food products by liquid chromatography. The saponification and extraction procedure has been optimized in terms of a significant reduction in quantities of chemical reagents and samples used, as well as modified conditions for conducting the saponification step. The introduced changes will lead to reduction in the costs of this analysis. Additionally, a clear advantage is the use of uncomplicated apparatus. The conditions of instrumental analysis were also reviewed. The developed method was validated by determining such parameters as the method linearity, recovery, precision, limits of determination, working range, and uncertainty. The validation procedure was carried out in accordance with the FDA guidelines for the validation of bioanalytical methods and the ICH technical requirements for the registration of medicinal products for human use [39,40].

Typically, studies on vitamins only concern the analysis of individual analytes separately. Only a few publications concern the examination of both vitamins A and E simultaneously. Moreover, the comparison with other publications shows that an individual method of sample preparation should be checked for each matrix. In this work, a method of simultaneous determination of both vitamins in three completely different matrices (spinach, almonds, and cheese) was tested. Therefore, four different methods of sample preparation for analysis were examined and the most favorable one was selected for the indicated matrices. In the study conducted by Shim et al. [45], determination of vitamins A and E in powdered milk and eggs was done. The sample preparation consisted in saponification in the presence of 1 M KOH with the addition of NaCl and butylated hydroxytoluene (BHT), the process was carried out at 100 °C for 30 min, and extraction was carried out with petroleum ether. Good linearity was achieved in the studied range during determination of vitamins A and E with ultra-HPLC. The regression analysis had shown good determination coefficient (R^2^), from 0.9984 to 1.0000. LOD and LOQ were 0.015 mg/kg and 0.045 mg/kg, respectively, for retinol, and 0.014 mg/kg and 0.042 mg/kg, respectively, for α-tocopherol. Furthermore, Katsa et al. [33] analyzed fat-soluble vitamins (A, D3, E) in rice-based baby products using two different chromatography techniques. The method described by these authors was based on enzymatic hydrolysis with α-amylase, saponification with ethanol, KOH, BHT, and ascorbic acid (at 80 °C for 30 min), and followed by extraction with petroleum ether. For analyzed vitamins, LOD and LOQ values were much lower for UHPLC-APCI -MS/MS than for HPLC-DAD, in which LOD was at a level of 0.98 µg/L and 5.4 mg/L and LOQ was recorded at a level of 2.9 µg/L and 16 mg/L for retinol and tocopherol, respectively. The recovery of the tested compounds was within the range of 85–107% for vitamin A and 92.5–103% for vitamin E. The recovery analyses conducted as a part of this study were determined for two concentration levels (retinol: 1 and 200 μg/g, and tocopherol: 1 and 500 μg/g). On a basis of obtained results a high level of recovery was found for analytes, of 98.8–110.1% for retinol, and 70.6–143.2% for tocopherol. The overestimated value (143.2%) of recovery occurred only in the case of vitamin E in one matrix (Parmesan cheese) at a higher level of enrichment. The recovery exceeded the suggested range (70–120%), while in the other two matrices, the recoveries were within the required range. In the case of samples with a very high fat content, a higher methanol volume or a lower sample weight can be used, to improve this parameter [28,29]. The method developed by Irakli et al. [46] used for simultaneous determination of lipophilic antioxidants, demonstrated the recovery ranging from 93.9 to 101.8% for tocopherol analogues. The use of HPLC-DAD by Katsa et al. [33], demonstrated recoveries within a range of 92.5–103% for α-tocopherol, and 85–107% for retinol. On the other hand, the study conducted by Rathi et al. [47] demonstrated recoveries of 61.86–116.9% for all examined analytes (retinol, lutein, and β-carotene).

The precision value expressed as a coefficient of variation (CV) in the proposed method was 6.5–8.9% for vitamins E and A, and definitely meets the FDA and ICH guidelines in this respect (CV ≤ 15%) [39,40]. Other authors in their works focusing on determination of these compounds achieved the precision value below 5% [48,49,50] both for retinol and for tocopherol.

In order to verify the suitability of the developed method for the specified purpose, it was used to determine vitamins A and E concentrations in real samples. The practicality of the proposed technique was indicated by the results obtained for 15 real samples tested (vegetables, nuts, and cheeses). The results obtained for the analyzed food products, expressed as α-tocopherol and retinol concentrations, are shown in Table 1. The results obtained for the analyzed actual samples were compared to vitamins A and E content in relevant food products according to the United States Department of Agriculture [19] and literature data [44].

The vitamin A content was the highest (1049.75 μg/100 g of the product) in the green parsley sample. In Parmesan cheese, the retinol content was nearly half of that value (563.22 μg/100 g of the product). In the case of vitamin E, its highest concentration was found in the pumpkin seeds sample (7606.22 μg/100 g of the product), while in fresh spinach the α-tocopherol content was slightly lower (6230.7 μg/100 g of the product). The lowest retinol and α-tocopherol content was noted in samples of nuts, of 73.9 μg/100 g of the product and of 1023.11 μg/100 g of the product for walnuts and cashews, respectively. The work of Kornsteinera et al. [51] presents an analysis of tocopherols in 10 different types of nuts; where they were not found in macadamia nuts. The highest average α-tocopherol content was found in hazelnuts (3.4 mg/100 g of oil).

A comparison with available reference data for food products showed both certain similarities and discrepancies with our results. The reference data for the α-tocopherol content in dill and cashew samples were similar to the results shown in Table 1. Furthermore, a sample of almonds showed the retinol content of 95.20 μg/100 g of the product, where this value is about 158 times higher than provided in the literature (0.6 µg/100 g). Vitamins content in food products may differ depending on a country of origin, storage, and processing, and properties of vitamins A and E, such as their sensitivity to light, temperature, and oxygen, may cause problems with their determination in the future [52]. Detailed quantitative measurements of vitamins provide a reliable evaluation of the product quality and an option to monitor their recommended daily intake [53].

On the basis of comparative assessments of analytical methods available in the literature for the determination of vitamins A and E, it can be concluded that this method is effective, accurate, and useful for routine quantitative analyzes of fat-soluble vitamins.

## 4. Materials and Methods

### 4.1. Chemicals and Reagents

Methanol and *n*-hexane of high purity were purchased from Honeywell Specialty Chemicals Seelze GmbH (Hannover, Germany). L-ascorbic acid and anhydrous sodium sulfate were obtained from Chempur (Piekary Śląskie, Poland), and potassium hydroxide was purchased from POCH (Zabrze, Poland). Water for sample preparations was purified through SolPure XIO P (ELKAR, Kęty, Poland).

### 4.2. Preparation of Standard Solutions

The individual vitamin analytical standards were obtained from Sigma Aldrich (Taufkirchen, Germany). Both standards were of >98% purity, and they were prepared in concentrations of about 2000 µg/mL in methanol and stored in closed dark glass bottles at ≤−16 °C to prevent degradation. Then, the stock solutions were used to prepare working solutions of vitamins A and E in methanol through a series of dilutions to determine 6-point calibration curves in the concentration range of 1.0 to 500 μg/mL (retinol) and of 1.06 to 532 μg/mL (α-tocopherol). The prepared standards were also used for sample fortification in validation experiments. All working solutions of vitamin standards were stored at 4 °C in dark pending the analysis, for up to one month.

### 4.3. Sample Preparation Procedure

The samples were obtained commercially from food markets. Three representative matrices were selected for the tests, of animal (Parmesan cheese) and plant (almonds and spinach) origin. The initial content of vitamins A and E in the tested samples was taken into account in the validation studies. The sample extract was prepared in the following steps: isolation of vitamins in the process of saponification, extraction, concentration, and appropriate dilution of the analytes.

The method for preparing samples for the vitamins A and E analyses in food products by liquid chromatography with ultraviolet-visible detection was a modification of standard methods available in the literature [29,31,50,54]. The proposed solution consists of a significant reduction in the weight of the tested products and quantities of reagents added at the saponification stage and also minimizing the amount of solvent used for extraction.

A total of 0.5 g of the tested product was weighed into glass screw-cap test tubes (15 mL), then 0.1 g of L-ascorbic acid, 5 mL of methanol, and 1.5 mL of 50% KOH solution were added. For the validation samples, standards were added at two concentration levels, 1 μg/mL and 200 μg/mL, and 1 μg/mL and 500 μg/mL for vitamins A and E, respectively. The samples were mixed, protected from light, and then four different sample preparation approaches were used: (i) left to “saponify overnight” (18 h) at a room temperature (21 °C), (ii) placed in a water bath at 80 °C for 30 min, (iii) placed in an ultrasound water bath at 60 °C for 25 min; and (iv) in an ultrasound water bath at 74 °C for 70 min. The saponified sample was quantitatively transferred to a separatory funnel and extracted with 10 mL of n-hexane vigorously for 2 min. The hexane extraction was repeated 3 times, and the combined hexane extracts were washed in a separatory funnel 3 times with 20 mL of distilled water until the pH was neutral. Then, residual water from the sample was removed by filtering the sample extract through anhydrous sodium sulfate filter. The resulting sample extracts were evaporated to dryness under nitrogen. The residues were then dissolved in 1 mL methanol, filtrated through a 0.45 μm Teflon filter, and transferred into 2 mL amber HPLC vials. Full validation was performed for the best method.

### 4.4. Instrumentation and Chromatographic Conditions

Determination of the retinol and α-tocopherol content was carried out by high-performance liquid chromatography with UV-VIS/DAD detection (Dionex, model Ultimate 3000, Germering, Germany) in the reverse phase, in the isocratic elution mode. The mobile phase flow rate was 1 mL/min and the injection volume was 20 μL. Sample separation was carried out on an ACE 5 C18 column packed with 5 μm (250 mm × 4.6 mm) shell particles with precolumn. The spectrophotometric detection for vitamin determination was performed at λ = 325 nm and 290 nm for vitamins A and E, respectively. The data was processed with Chromeleon 6.8 software (Dionex Corporation, Sunnyvale, CA, USA). Optimization of chromatographic conditions was carried out using different proportions of mobile phase components: methanol–water (86:14; 95:5; 100:0 (*v*/*v*)) at the flow rate of 1 and 1.2 mL/min, as well as at different temperatures of the chromatographic column oven (25 °C and 30 °C). The total analysis time was 16 min.

### 4.5. Validation Studies

Validation studies were conducted to determine the suitability of the developed method for three representative matrices: Parmesan cheese, spinach, and almonds. The following validation parameters were determined: linearity, limit of quantification (LOQ), limit of determination (LOD), repeatability/precision, accuracy, and method uncertainty. The validation of the method was carried out in accordance with the current the FDA guidelines, i.e., guidelines for the validation of bioanalytical methods [40] and technical requirements for the registration of medicinal products for human use [39]. The working range and linearity of the method were estimated using a range of concentration values determined with the assumed precision, accuracy, and uncertainty. Linearity was studied by HPLC-UV-VIS/DAD analysis at six different concentration levels over the ranges of 1.00–500 μg/mL (retinol) and 1.06–532 μg/mL (α-tocopherol), and they were applied in three repetitions per level over three days. The linear equations of the curve and the coefficient of determination (R^2^) were established. The coefficient of determination R^2^ ≥ 0.99 was adopted as the acceptance criterion. The LOQ was determined by performing recovery tests at the lowest enrichment level (1.00 μg/g) for which the recovery and precision values met the validation requirements. The LOD was determined on the basis of the analyte signal-to-noise ratio of the baseline, and a 3:1 ratio was adopted as the LOD value. In the recovery experiments, samples were spiked with the appropriate volumes of working standard solutions of vitamins, at two levels: LOQ, and 200 × LOQ (for retinol) and 500 × LOQ (for α-tocopherol). The accuracy of the method was assessed by analyzing the spiked samples tested and calculating recovery and average recovery. The method is considered accurate if the average recovery obtained over the series of measurements is in the range of 70–120%. Repeatability/precision was assessed by recovery studies on a series of samples (n = 3) spiked at two concentration levels. Subsequently, on the basis of the obtained series of results, the standard deviation (S) and the coefficient of variation (CV) were determined. The acceptance criterion was CV ≤ 15%. Measurement uncertainty (U) was estimated on the basis of the results obtained in the validation process, where the main sources of uncertainty were the repeatability of recovery from spiked samples and the uncertainty of the mean recovery calculated on the basis of rectangular distribution. The expanded relative uncertainty was calculated using the coverage factor k = 2 at the 95% confidence level [41,42,43].

## 5. Conclusions

In this article, the sample preparation procedure (based on saponification and extraction) was optimized to separate vitamins A and E from three representative matrices and analyze them using the HPLC-UV-VIS/DAD system. A novelty in these studies was the significant reduction in quantities of reagents used (down to 30 mL of hexane and 20 mL of water) at the extraction stage and in the saponification process: a reduction in the sample weight to 0.5 g, the quantity of ascorbic acid (to 0.1 g), and a volume of methanol and potassium hydroxide to 5 mL and 1.5 mL, respectively. In consequence, it will enable a reduction in the costs of conducting analyzes for the determination of these substances. The conditions of conducting the saponification stage were also optimized, and the tests showed the best validation parameters for the process carried out at room temperature for 18 h. The method was validated jointly for both vitamins. General linearity parameters, precision, and accuracy were highly satisfactory, with the exception of recovery for Parmesan cheese at a higher spiking level. The extended uncertainty of the method was also excellent. The proposed analytical procedure is efficient, accurate, and repeatable, making it suitable for the simultaneous determination of retinol and alpha-tocopherol in food products. The developed method was successfully used to test a total of 15 real food samples of plant and animal origin. The results of the analysis of the samples revealed the presence of the tested compounds in almost all samples. Nevertheless, the obtained results indicate the need for further monitoring of these vitamins.

## Figures and Tables

**Table 1:** Vitamins A and E analyses in real food samples.

Product	The Concentration of Vitamin E (µg/100 g)	The Concentration of Vitamin E According to Literature (μg/100 g)	The Concentration of Vitamin A (µg/100 g)	The Concentration of Vitamin A According to Literature (μg/100 g)
Arugula	2670.7	500.0 [19]	n.d	711 [19]
Frozen spinach	4308.4	2897.4 [19]	80.33	3630 [19]
Fresh spinach	6230.7	2033.3 [19]	n.d	2814 [19]
Dill	1775.6	1800.0 [44]	n.d	2316 [19]
Parsley leaves	4370.7	750.9 [19]	1049.75	2526 [19]
Parmesan	1445.8	530.0 [19]	563.22	262 [19]
Cheese “Złoty Mazur”	1360.0	598.5 [19]	430.44	372 [19]
Cheese “Radamer”	1383.1	598.5 [19]	106.39	372 [19]
Mozzarella	1114.2	187.5 [19]	430.77	296 [44]
Cheese “Edam”	1144.0	246.9 [19]	466.35	367 [44]
Almond	5374.22	26,100 [44]	95.2	0.6 [19]
Cashew	1023.11	900 [19]	179.54	n.d. [19]
Walnut	1840.89	6050 [44]	73.9	6 [19]
Walnut	1251.56	6050 [44]	254.28	6 [19]
Pumpkin seed	7606.22	2180 [19]	n.d	50 [19]

n.d—not detected.

## Data Availability

Available on request and with regulations.

## Supplementary Materials

The following supporting information can be downloaded at: https://www.mdpi.com/article/10.3390/molecules28083449/s1, Figure S1: Chromatogram of the stock solution of mixed standards and absorption spectra: retinol (concentration—200 µg/mL, Rt—5.573 min.) and α-tocopherol (concentration—500 µg/mL, Rt—13.139 min); Figure S2: Chromatogram for the real samples: (a) green parsley, (b) pumpkin seeds, (c) cheese “Złoty mazur”.

## Sample Availability

Samples of the compounds are not available from the authors.
