# Supplementary material for: A Miniaturized Sample Preparation Method for the Determination of Vitamins A and E in Food Products
Source: Molecules. 2023 Apr 13;28(8):3449. doi: 10.3390/molecules28083449 (PMC10144259; doi:10.3390/molecules28083449)

molecules

MDPI

**Supplementary Material**

Figure S1. Chromatogram of the stock solution of mixed standards and absorption spectra: retinol (concentration - 200 μg/mL, Rt - 5.573 min.) and α-tocopherol (concentration - 500 μg/mL, Rt - 13.139 min.)

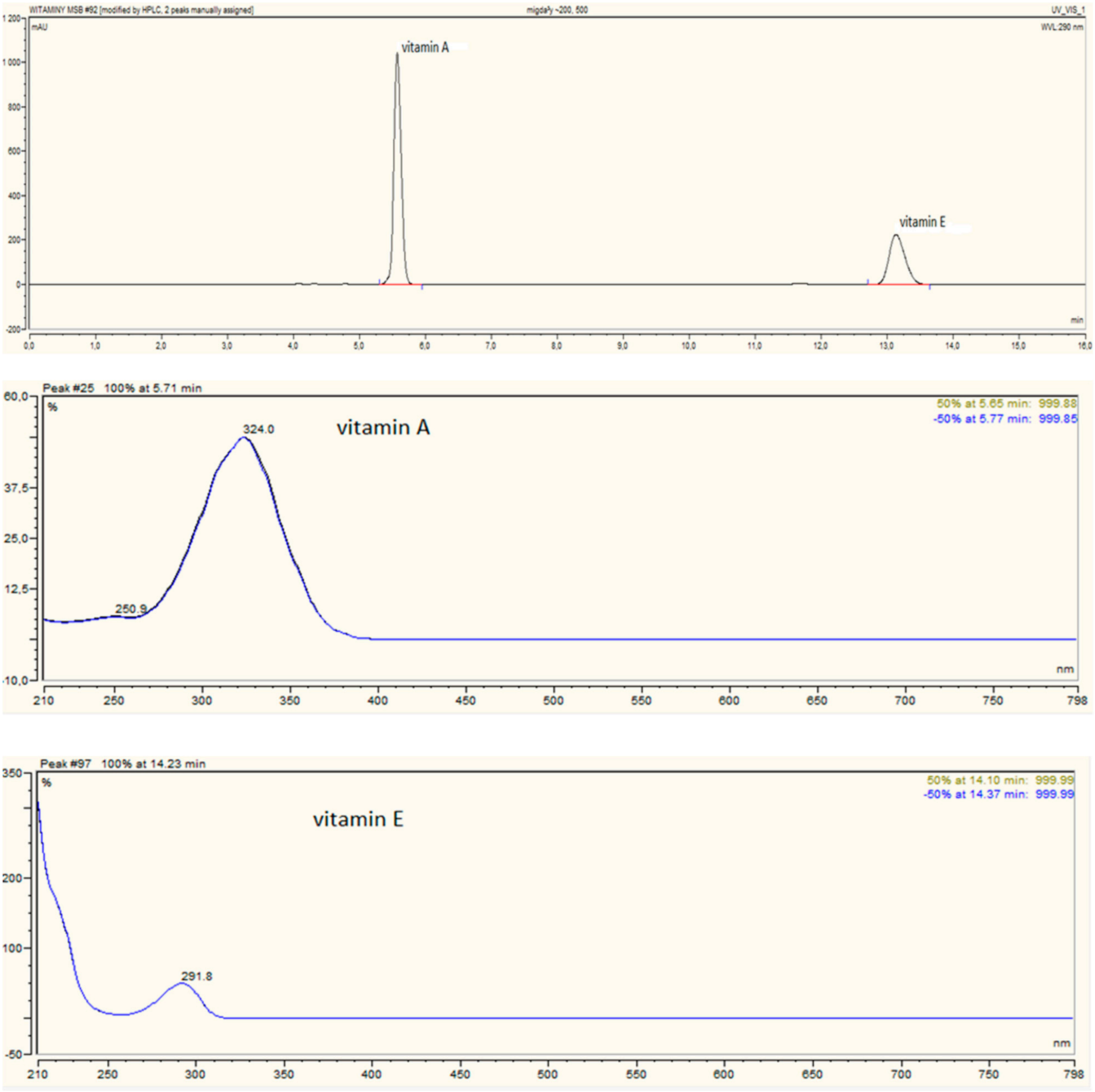

Figure S2. Chromatograms for the real samples: a) green parsley, b) pumpkin seeds, c) cheese “Złoty mazur”

a)

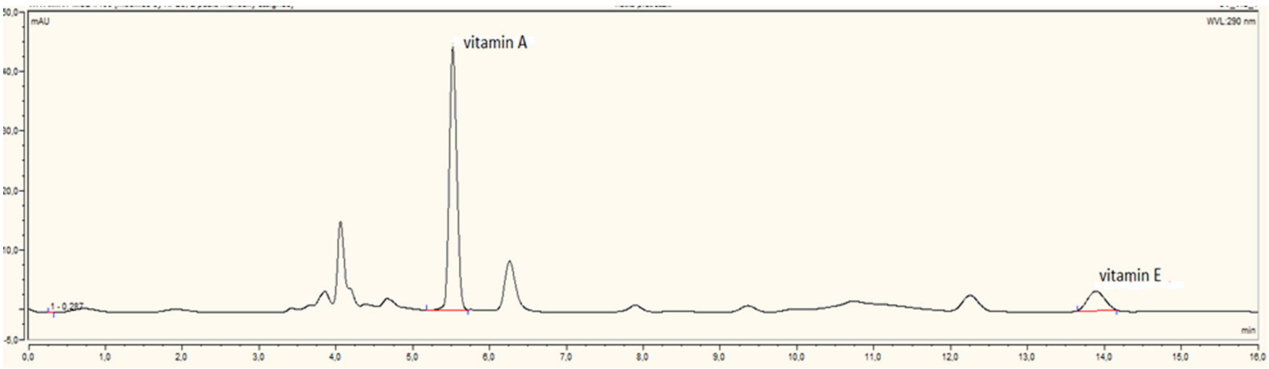


b)

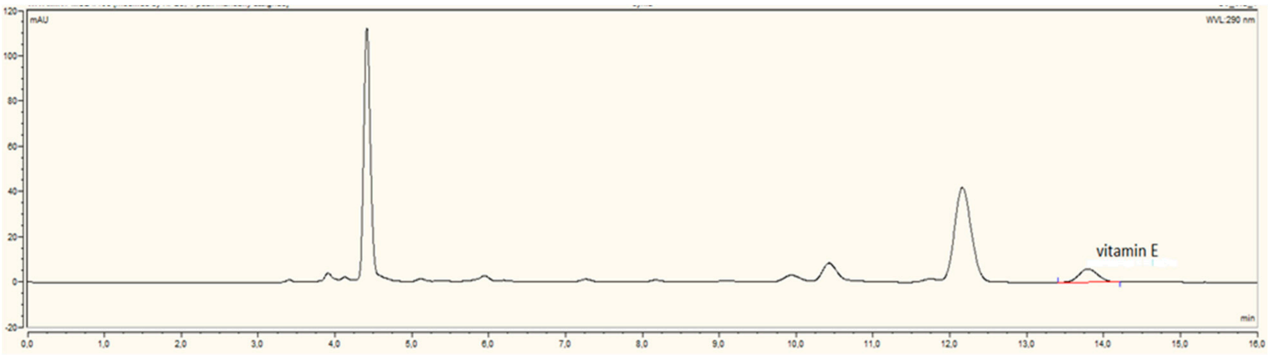


c)

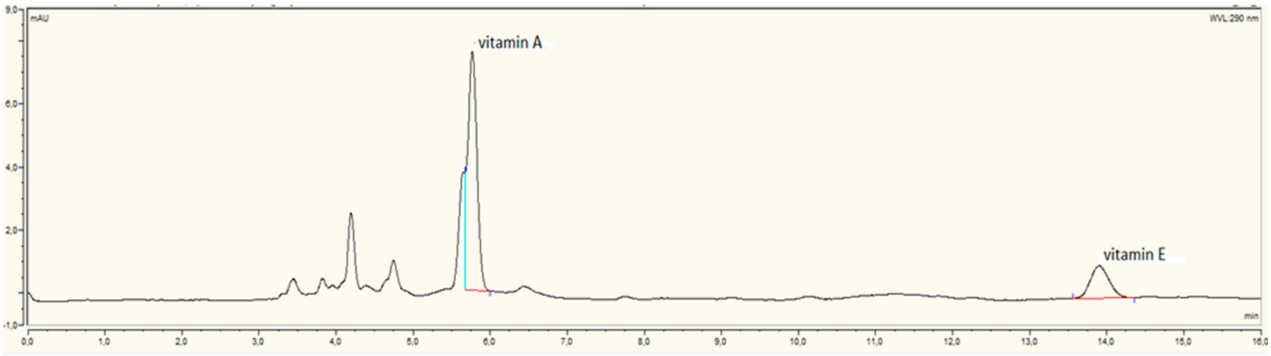

Supplement: Supplementary file 1 [file molecules-28-03449-s001.zip › molecules-2310065-supplementary.pdf]
